# Editorial: Vaccines and immunization services during the pandemic era and beyond

**DOI:** 10.3389/frhs.2024.1394381

**Published:** 2024-03-14

**Authors:** Edina Amponsah-Dacosta, Alma Fulurija, Anthony Afum-Adjei Awuah, Smriti Mathema, Oghenebrume Wariri

**Affiliations:** ^1^ Vaccines for Africa Initiative, School of Public Health, Faculty of Health Sciences, University of Cape Town, Cape Town, South Africa; ^2^ Telethon Kids Institute and School of Biological Sciences, University of Western Australia, Perth, WA, Australia; ^3^ Global Health and Infectious Diseases Research Group, Kumasi Centre for Collaborative Research in Tropical Medicine, Kumasi, Ghana; ^4^ Research Group Global One Health, Department of Implementation Research, Bernhard Nocht Institute of Tropical Medicine, Hamburg, Germany; ^5^ Department of Molecular Medicine, School of Medicine and Dentistry, Kwame Nkrumah University of Science and Technology, Kumasi, Ghana; ^6^ Department of Pediatrics, Kathmandu Medical College Teaching Hospital, Kathmandu, Nepal; ^7^ Vaccines and Immunity Theme, MRC Unit The Gambia at London School of Hygiene and Tropical Medicine, Fajara, The Gambia; ^8^ Department of Infectious Disease Epidemiology, Faculty of Epidemiology and Population Health, London School of Hygiene and Tropical Medicine, London, United Kingdom

**Keywords:** COVID-19, equity, vaccines, vaccine preventable diseases, immunization, pandemic

**Editorial on the Research Topic** Vaccines and immunization services during the pandemic era and beyond

It is now well established that the COVID-19 pandemic caused by the SARS-CoV-2 virus significantly impacted immunization services, threatening to reverse the substantial gains made in the prevention and control of vaccine preventable diseases, globally. Consequently, an estimated 23–25 million children missed one or more of their scheduled vaccine doses at the height of the pandemic (1, 2). Foremost on the global health agenda is re-prioritizing immunization services to recover vaccine coverage rates and secure population health and well-being. While recovery is ongoing, progress has been suboptimal or inconsistent across countries. Robust interrogations into the disruptions caused by the pandemic at country and community levels are required to draw from lessons learned in enhancing the resilience and responsiveness of immunization systems globally. Paradoxically, the pandemic has also been a catalyst for innovations in the vaccine space. We are seeing a substantial shift in the vaccine landscape, from increased interest in vaccine discovery and development, to how policies are formulated and implemented, and how we conduct vaccine research and deliver immunization services (3–5).

To chart a sustainable way forward in the face of current epidemics and future pandemics, immunization services will need to be guided by reliable evidence generated to suit local contexts. Our goal for this research topic therefore was to collect a rich diversity of articles documenting how the COVID-19 pandemic influenced vaccines research and immunization services, with a focus on recovery and strengthening efforts at all levels and across the vaccinology cascade (from vaccine development through to policy formulation and access/delivery of essential vaccines, as well the barriers to implementation like vaccine hesitancy). The scope and themes of interest included, but were not limited to, original research articles, brief research reports, study protocols, as well as opinion and perspective pieces covering the following topics:

1. Pandemic prevention and preparedness with a focus on innovations and advancements in the field;
2. Equitable access to vaccines and immunization services for underserved populations. These include hard-to-reach or hard-to-vaccinate populations, adolescents, pregnant women, the elderly, marginalized persons, and migrant populations;
3. Vaccine confidence, hesitancy, and acceptability;
4. Vaccine communication and demand generation;
5. Leveraging lessons learned from COVID-19 control efforts to improve prevention and control of existing and emerging vaccine preventable diseases;
6. Expansion of vaccinology expertise to support scale-up of immunization programs, particularly in low- and middle- income countries;
7. Advances in evidence-informed policy- and decision- making for vaccines and immunization services;
8. Implementation and integration of immunization programs into primary health care systems; and
9. The economics of vaccine preventable diseases, vaccines, and immunization services.

Overall, vaccine (in)equity emerged as a prominent theme across the collection of articles in this research topic, with a focus on advocating for inclusive, responsive, and fair access to immunization services. As a primary example, COVID-19 mitigation strategies including vaccination programs, have not always been responsive to the specific needs of underserved and marginalized populations. This was the case in a study conducted in Germany to assess the determinants of COVID-19 vaccine acceptance and access among people experiencing homelessness. In this study, Grune et al. found that vaccine acceptance within this population was influenced by their confidence in the vaccine, as well as the political and healthcare system. Their individual COVID-19 risk perceptions and a sense of collective responsibility also played a role. Carol and Amro found that inter-group dynamics and boundaries, as well as entrenched binary perceptions of “us” vs. “them” played a significant role in how minority groups (Bedouins and internally displaced Palestinians) and majority groups (non-refugees or non-Bedouins) living in the West Bank, prioritized COVID-19 booster vaccination. These dynamics can have potential negative consequences for the healthcare of minorities. In a study conducted in Slovakia by Filakovska Bobakova et al. marginalized Roma communities were reported to experience significant barriers when accessing vaccination services. These barriers include limited or disparate coverage by medical insurance companies for vaccines like the human papillomavirus (HPV) vaccine, health worker shortages, impaired relationships between health workers and Roma communities, and poor access to appropriate risk communication and health information. Improving fair and just access to immunization services for underserved populations should be a top priority in the public health agenda. This can be achieved through policy reforms and innovative interventions which carefully consider the lived experiences of specific population groups. In line with this, Broach et al. detail how a novel vaccine delivery model, known as the Mobile Vaccine Equity Enhancement Program, successfully improved the rapid and equitable delivery of the COVID-19 vaccine among communities with high social vulnerability indices in Central Massachusetts. Similarly, Skaathun et al. propose Project 2VIDA! which is a community-based participatory research intervention aiming to address key barriers to access and acceptance of COVID-19 vaccines among African American and Latino communities living in Southern California. Taken together, these innovative interventions could provide crucial learnings for enhancing equitable delivery of immunization services for persistently underserved communities.

The contributions of vaccine inequity, or better yet vaccine apartheid, to growing sentiments of public mistrust in COVID-19 vaccines specifically, and vaccine hesitancy more broadly, during the pandemic era cannot be overstated. Nkole et al. offer pivotal perspectives on the importance of community experiences in better understanding how inequitable vaccine supply undermines demand generation, especially in the African context. Drawing on lessons learned from the COVID-19 experience, Nkole et al. further suggest the need for more equitable emergency response strategies, improved accountability of global health partners and relevant stakeholders, and the importance of applying a human rights-based approach to vaccine delivery, grounded in key principles such as equity, transparency, and community. To counter vaccine hesitancy and build back trust in immunization services, several countries have explored a myriad of interventions. One such intervention is the COVID-19 Vaccine Communication Campaign (CVCC) instituted by the Chinese state. A major finding of an evaluation conducted by Yang and Han to assess the vulnerabilities of the CVCC was the influence of top-down political pressure, leading the authors to propose broader stakeholder engagements and optimization of service provision to de-politicize COVID-19 vaccination programs if a successful vaccine communication campaign is to be achieved.

There were some useful insights into COVID-19 vaccine implementation strategies from various countries. Chen et al. evaluated selected COVID-19 vaccine clinics in the United States and found that sound communication systems, multidisciplinary leadership structures, and adoption of patient-centered engagement strategies were some of the strong drivers of implementation while vaccine scarcity posed significant challenges. In Nigeria, a government-sanctioned family-centered approach to increasing uptake of COVID-19 vaccines, known as the Whole Family Approach, showed promising findings. Offor et al. describe how this unique health promotion intervention draws on the high demand for other primary health services (e.g., malaria, diabetes, hypertension, and reproductive services) among families in Nigeria to increase demand for COVID-19 vaccines and routine immunization services in general. Recognizing the fact that trypanophobia or fear of needles could contribute to COVID-19 vaccine refusal or hesitancy, Wang et al. conducted a cross-sectional survey in China to assess perceptions and willingness towards other prospective modes of vaccine administration. Interestingly, the overwhelming majority indicated a preference for intramuscular injection compared to oral inhalation or intranasal spray, although the findings may have been influenced by a low level of awareness about alternative routes of vaccine administration currently undergoing clinical trials (6).

It is also worth highlighting how other routine immunization services were impacted during the COVID-19 pandemic. Acute or prolonged disruptions have been observed more frequently in countries with suboptimal pre-pandemic vaccine coverage rates compared to those with stable immunization systems (1, 7–9). In a health facility-based study conducted in South Africa, Manan et al. found that routine childhood vaccine coverage rates fell below national targets with uncertainties about the risk of COVID-19 contributing to low clinic attendance. Positive vaccine seeking behavior was observed among caregivers with good family support and those who were beneficiaries of the national social welfare grant scheme. Such findings are critical to informing context-specific interventions aimed at generating vaccine demand. Anraad et al. in their paper detail the development of an online tailored decision aid coupled with an intervention to promote informed decision making on pertussis vaccination among pregnant women in the Netherlands. This intervention was informed by a preliminary needs assessment which showed that pregnant women tend to base their decisions on vaccinating during pregnancy on information accessed online in addition to discussions with their healthcare providers and social contacts. Given the heavy presence of anti-vaccine sentiments online bolstered during the COVID-19 pandemic, such intervention could only serve to reduce the devastating impact of mis- and dis-information on health outcomes. In Indonesia, the COVID-19 pandemic was reported to have dramatically impacted the performance of routine immunization services. Here, private health facilities were found to be adequately staffed, had fewer vaccine stock-outs, and provided sufficient time for essential childhood immunization services. As such, Suwantika et al. call for better coordination between public and private sectors, and an expansion of the role of private healthcare facilities in order to improve the performance of the national routine immunization program. The successful integration of immunization and other primary health services in Lebanon, amidst multiple, nested crises such as the COVID-19 pandemic, the economic collapse, fuel crisis, Beirut blast, and a large refugee presence, was attributed by Kapuria et al. to strong partnerships between government institutions and global health agencies.

Despite having the highest burden of cervical cancer, African countries experience significant challenges in implementing life-saving HPV vaccination programs, suggesting underlying health systems constraints which have been exacerbated by the COVID-19 pandemic (10)*.* In Kenya, acceptance and uptake of the HPV vaccine has been negatively impacted by growing vaccine hesitancy at the community level following the COVID-19 pandemic. Umutesi et al. propose a study to assess barriers and facilitators to HPV vaccine delivery, and the acceptability of a single-dose strategy. It is anticipated that the findings will provide useful insights into the single-dose strategy aimed at enhancing uptake of the HPV vaccine among adolescent girls in Kenya. Strategies aimed at scaling-up HPV immunization programs should be informed by the perceptions and opinions of adolescent girls themselves. To increase their knowledge and awareness of HPV vaccination and thereby improve acceptance and uptake, adolescents in Zambia who participated in a study by Lubeya et al. suggest making vaccine information more accessible within communities through social mobilization campaigns and school curricula, and also stress the importance of the active involvement of politicians in the country.

Finally, as Manga et al. point out, a well-trained workforce is crucial to getting immunization programs back on track for those who need them the most. In their article, the authors report on a proof-of-concept study assessing the training needs of alumni (policy makers, programme managers, immunization providers, and scientists working in the field of vaccinology) of the Annual African Vaccinology Course who required refresher training because of the rapid evolutions in the field and the challenges brought on by the COVID-19 pandemic. By tailoring a vaccinology webinar series to meet these needs, the authors were able to show the success of implementing a low-cost, widely accessible continuous health education program in the African context.

This research topic comprises 18 articles which contribute highly researched and thought-provoking findings on how the COVID-19 pandemic has influenced the vaccines and immunization landscape across various contexts, globally. These articles are an important contribution to the growing body of evidence required to inform immunization recovery strategies in the pandemic era and beyond. We anticipate that this research topic will stimulate further dialogue and inspire future research aimed at “pandemic-proofing” immunization services.
